# Efficacy and safety of thrombopoietin receptor agonists in children with chronic immune thrombocytopenia: a meta-analysis

**DOI:** 10.18632/oncotarget.23487

**Published:** 2017-12-19

**Authors:** Jian-Chun Guo, Yi Zheng, Hai-Tao Chen, Haixia Zhou, Xian-Hui Huang, Li-Ping Zhong, Huai-Bin Zhou, Yu Huang, Dan-Li Xie, Yong-Liang Lou

**Affiliations:** ^1^ Department of Microbiology and Immunology, School of Laboratory Medicine, Wenzhou Medical University, Wenzhou, Zhejiang 325035, China; ^2^ China Ministry of Education Key Lab of Laboratory Medicine, Wenzhou, Zhejiang 325035, China; ^3^ Department of Hematology, The Second Affiliated Hospital and Yuying Children's Hospital of Wenzhou Medical University, Wenzhou, Zhejiang 325027, China; ^4^ Department of Orthopaedic Surgery, Zhongnan Hospital of Wuhan University, Wuhan, Hubei 430071, China; ^5^ Department of Infectious Diseases, The First Affiliated Hospital of Wenzhou Medical University, Wenzhou Key Laboratory of Hepatology, Hepatology Institute of Wenzhou Medical University, Wenzhou, Zhejiang 325025, China

**Keywords:** thrombopoietin receptor agonists, immune thrombocytopenia, children, meta-analysis

## Abstract

**Background and Aim:**

Thrombopoietin receptor agonists (TPO-RAs) have been shown to be safe and effective for adults with chronic immune thrombocytopenia (ITP). The aim of this meta-analysis is to assess the efficacy and safety of thrombopoietin receptor agonists for children with chronic ITP.

**Materials and Methods:**

Clinical randomized controlled trials (RCTs) evaluating the efficacy and safety of TPO-RAs in pediatric ITP patients published up to June 2017 were retrieved from PubMed, Cochrane Library, and Embase databases. Relevant data were extracted, and the Physiotherapy Evidence Database scale was used to assess the methodological quality. Stata/SE 12.0 was used to perform a meta-analysis.

**Results:**

Seven RCTs were included, with 238 patients and 107 patients in the TPO-RA group and the control group, respectively. Assessing efficacy, better results were found in the TPO-RA group for the rate of overall platelet response, durable response, and rescue medication needed. Furthermore, the TPO-RA group yielded superior results in the incidence of clinically significant bleeding events but had a comparable result in the incidence of any bleeding events and severe bleeding events. No significant difference was found between the two groups in health-related quality of life and parental burden. Assessing safety, no significant difference was found between the two groups in the incidence of any adverse events and severe adverse events.

**Conclusions:**

TPO-RAs are effective and safe agents for the treatment of chronic ITP in pediatric patients. Eltrombopag appears to be better than romiplostim in terms of the rate of rescue medication needed and clinically significant bleeding events.

## INTRODUCTION

Immune thrombocytopenia (ITP), previously known as idiopathic thrombocytopenia purpura, is an autoimmune disorder characterized by accelerated platelet destruction as well as defective platelet production [1–4]. Patients with persistently low platelet counts are often at a high risk for bleeding, ranging from bruising, petechiae, to clinically significant bleeding, even intracranial hemorrhage [1, 5–7]. Although ITP in most children is usually a benign condition that they can spontaneously recover from within half a year to one year [8, 9], approximately 5–15% of pediatric patients with chronic ITP still require special treatment [1, 8–10]. Meanwhile, chronic ITP in children can negatively affect their health-related quality of life (HRQoL) and impose a burden on their parents [8, 11, 12]. Thus, the primary goal of treatment for this disorder is to prevent bleeding by increasing the platelet count to a stable, safe level with the fewest possible treatment-associated adverse effects [8, 13–15].

Guidelines from the American Society of Hematology [16] and International Consensus Report [17] for the treatment of symptomatic ITP in children recommend the use of intravenous immunoglobulin, prednisone, or anti-D immunoglobulin as first-line treatments and the use of rituximab, dexamethasone, or splenectomy as treatments for persistent or chronic ITP [4]. However, the side effects and potential short-term and long-term risks of these interventions can be problematic [7, 18–23]. Splenectomy, an invasive procedure, is unacceptable to many families because its postoperative complications, especially sepsis, remain a concern that probably persists for life [24–26]. While splenectomy has been routinely used for decades with good success, the potential risks and irreversibility of treatment has reduced its use, particularly in pediatric patients [27].

Thrombopoietin (TPO) is a relatively lineage-specific cytokine that stimulates the proliferation and differentiation of megakaryocytes from committed progenitor cells, resulting in the regulation of platelet production [28]. Thrombopoietin receptor agonists (TPO-RAs) are the only therapies available that can specifically promote platelet production [5]. The two approved TPO-RAs, eltrombopag [13, 29, 30] and romiplostim [31], have achieved good results [29, 31, 32] and are recommended as second-line therapeutic options [16, 17] for adult ITP patients.

Eltrombopag has been recently approved for pediatric chronic ITP in the United States and Europe [5, 32]. However, there is still no evidence-based guideline on whether TPO-RAs are recommended for pediatric ITP patients [5, 6]. First, only a small fraction of all pediatric ITP patients will have chronic and/or refractory disease, so experience with treating such children is quite limited. Second, there are limited studies paying attention to TPO-RAs in pediatric ITP patients. Furthermore, it is still not clear whether the benefits of TPO-RAs can outweigh the potential risks associated with TPO-RAs in children. Thus, we conducted this meta-analysis to comprehensively evaluate the efficacy and safety of TPO-RAs in pediatric ITP patients.

## RESULTS

### Search results

**Figure 1:**
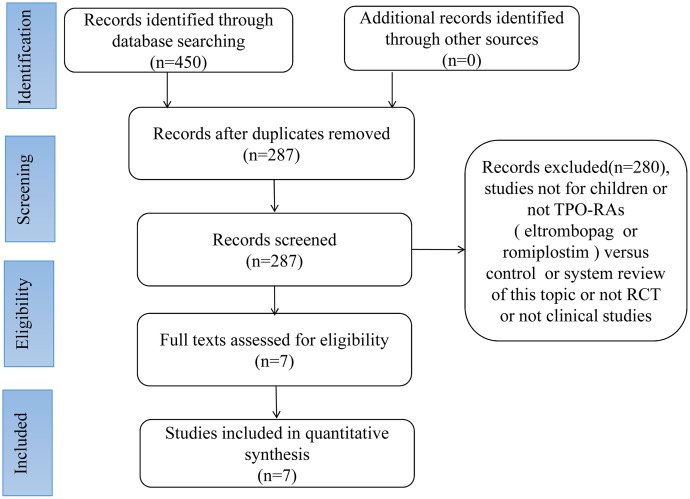
Flowchart of article selection process

A total of 450 relevant articles were initially selected according to the search strategy. Of these, 163 were excluded after checking for duplicates with the literature management software, Endnote X7. Next, 280 were excluded after reviewing the titles and abstracts. Finally, 7 articles [1, 4, 7, 8, 12, 18, 34] were included in the meta-analysis. A summary of the review process is presented in Figure 1.

### Description of included studies

All seven selected articles were written in English. These 7 articles compared the clinical outcomes of the TPO-RA group and control group in the therapy of ITP. No important difference was found between the two groups in age, sex, basic platelet count, and status of splenectomy. Follow-up periods in the included studies were ≥ 7 weeks. The total number of patients was 345: 238 patients and 107 patients were in the TPO-RA group and the control group, respectively. All basic article information is reported in Table 1, and the outcome measures of the 2 groups are reported in Table 2.

**Table 1:** Description of included trials

Study	Size (n)	Follow-up (weeks)	Age		Sex: F/M (n)		Baseline PC ´109/L		Duration of ITP (years)		Splenectomy (Y/N)		Outcomes
			TPO-RA	Control	TPO-RA	Control	TPO-RA	Control	TPO-RA	Control	TPO-RA	Control	
Tarantino et al. 2016	62	25	10 (6–14)	7.5 (6.5–13.5)	24/18	11/9	17·8 (7·5–24·5)	17·7 (9·8–24·1)	1.9 (1.0–4.2)	2.2 (1.5–3.7)	1/41	1/19	OR; DR; Rescue medication; Any bleeding events; Severe bleeding events; Severe AEs
Mathias et al. 2016	62	25	9.7 ± 4.1	9.4 ± 4.7	24/18	11/9	19.9 ± 19.3	17.5 ± 10.7	3.0 ± 2.8	3.0 ± 2.3	1/41	1/19	HqoL; Parental burden
Klaassen et al. 2012	22	13	9 (1–17)	11 (2–14)	4/13	2/3	13 (2–27)	9 (8–29)	2.4 (0.8–14.0)	4.1 (0.6–8.6)	6/11	2/3	HqoL; Parental burden
Elalfy et al. 2011	18	12	9.5 (2.5–16)	7 (4–15)	2/10	3/3	10.5(2–20)	10.5(6–20)	2.3 (1.2–7.0)	3.0 (1.5–6.5)	0/12	0/6	OR; Rescue medication; Severe bleeding events; All AEs; Severe AEs;
Bussel et al. 2011	22	12	9 (1–17)	11 (2–14)	4/13	2/3	13 (2–27)	9 (8–29)	2.4 (0.8–14.0)	4.1 (0.6–8.6)	6/11	2/3	OR; DR; Rescue medication; Any bleeding events; Severe bleeding events; Cilinical bleeding events; All AEs; Severe AEs
Grainger et al. 2015	92	12	9.4 (8.2–10.5)	9.8 (8.3–11.3)	30/33	14/15	< 30	< 30	3.4 ± 2.8	4.4 ± 3.4	4/59	0/29	OR; DR; Rescue medication; Any bleeding events; Cilinical bleeding events; All AEs; Severe AEs
Bussel et al. 2015	67	7	9 (8–10)	10 (8–12)	27/18	13/9	< 30	< 30	> 0.5	> 0.5	5/40	0/22	OR; DR; Rescue medication; Any bleeding events; Severe bleeding events; Cilinical bleeding events; All AEs; Severe AEs

F/M: female/male; PC: platelet count; ITP: immune thrombocytopenia; Y/N: yes/no; OR: overall platelet response; DR: durable response; HqoL: health-related quality of life; AEs: adverse events.

**Table 2:** Outcome measures of TPO-RA group versus control group

Study	N	OR (Y/N)		DR (Y/N)		Rescue medication (Y/N)		Any bleeding (Y/N)		Serious bleeding (Y/N)		Clinically significant bleeding. (Y/N)		HQoL		Parental burden		All AEs (Y/N)		Serious AEs (Y/N)	
		TPO-RA	Control	TPO-RA	Control	TPO-RA	Control	TPO-RA	Control	TPO-RA	Control	TPO-RA	Control	TPO-RA	Control	TPO-RA	Control	TPO-RA	Control	TPO-RA	Control
Tarantino (2016)	62	30/12	4/16	22/20	2/18	6/36	2/18	35/7	14/5	5/37	1/18			-	-	-	-		-	10/32	1/18
Mathias (2016)	62	-	-	-	-	-	-	-	-					80.2 ± 14.8	78 ± 18.9	53.7 ± 25.4	49.4 ± 18.2	-	-		
Klaassen (2012)	22	-	-	-	-	-	-	-	-					81.2 ± 13.3	75.5 ± 21.1	61.8 ± 21	29.8 ± 1.9	-	-		
Elalfy (2011)	18	10/2	0/6	-	-	1/11	2/4	-	-	0/12	2/4			-	-	-	-	6/6	3/3	0/12	0/6
Bussel (2011)	22	15/2	0/5	12/5	1/4	2/15	2/3	12/5	2/3	0/17	0/5	1/16	0/5	-	-	-	-	16/1	5/0	1/16	1/4
Grainger (2015)	92	47/16	6/23	25/38	1/28	12/41	7/22	45/18	20/9	-	-	3/60	2/27					51/12	21/8	5/58	4/25
Bussel (2015)	67	28/17	7/15	16/29	0/22	6/39	11/11	14/31	18/4	0/45	2/20	4/41	7/15					36/9	20/2	4/41	2/20

N : number of the patients; OR: overall platelet response; Y/N : yes/no; DR: durable response; HqoL: health-related quality of life; AEs: adverse events.

### Quality assessment results

All of the seven selected articles were RCTs and assessed using the PEDro scale. The results showed that all articles scoring ≥ 6 were of high quality. All seven studies used the randomization method. Three studies used concealed allocation. All studies demonstrated that their two groups were similar at baseline. All studies used a blinding method on their subjects, and six studies blinded their clinicians to group allocation. No studies used a blinding method on their assessors. All studies measured at least one key outcome from ≥ 85% of the subjects initially allocated to the groups. Point and distribution measurements were presented for at least one key outcome in all studies. The methodological score of each included RCT with general remarks is shown in Table 3.

**Table 3:** PEDro critical appraisal tool results

Study	Criteria											total
	1	2	3	4	5	6	7	8	9	10	11	
Tarantino et al.	✓	✓	✓	✓	✓	✓	×	✓	✓	✓	✓	9
Mathias et al.	✓	✓	×	✓	✓	✓	×	✓	✓	✓	✓	8
Klaassen et al.	✓	✓	×	✓	✓	✓	×	✓	✓	✓	✓	8
Elalfy et al.	✓	✓	×	✓	✓	×	×	✓	✓	✓	✓	7
Bussel et al.	✓	✓	×	✓	✓	✓	×	✓	✓	✓	✓	8
Grainger et al.	✓	✓	✓	✓	✓	✓	×	✓	✓	✓	✓	9
Bussel et al.	✓	✓	✓	✓	✓	✓	×	✓	✓	✓	✓	9

✓, satisfied criterion; ×, did not satisfy criterion.Criteria:

1. Eligibility criteria were specified.

2. subjects were randomly allocated to groups(in a crossover study, subjects were randomly allocated an order in which treatmentswere received).

3. allocation was concealed.

4. the groups were similar at baseline with.

respect to the most important prognostic indicators.

5. all subjects were blinded to the procedure.

6. all therapists who administered the therapy were blinded.

7. all assessors whomeasured at least one key outcome were blinded.

8. measures of at least one key outcomewere obtained from ≥ 85% of the subjects initially allocated to groups.

9. all subjects forwhom outcome measures were available received the treatment or control condition asallocated or, where this was not the case, data for at least one key outcome was analyzedby intention to treat.

10. the results of between-group statistical comparisons are reportedfor at least one key outcome.

11. the study provides both point measures and measures ofvariability for at least one key outcome.

### Efficacy analysis

### Primary outcomes

**Figure 2:**
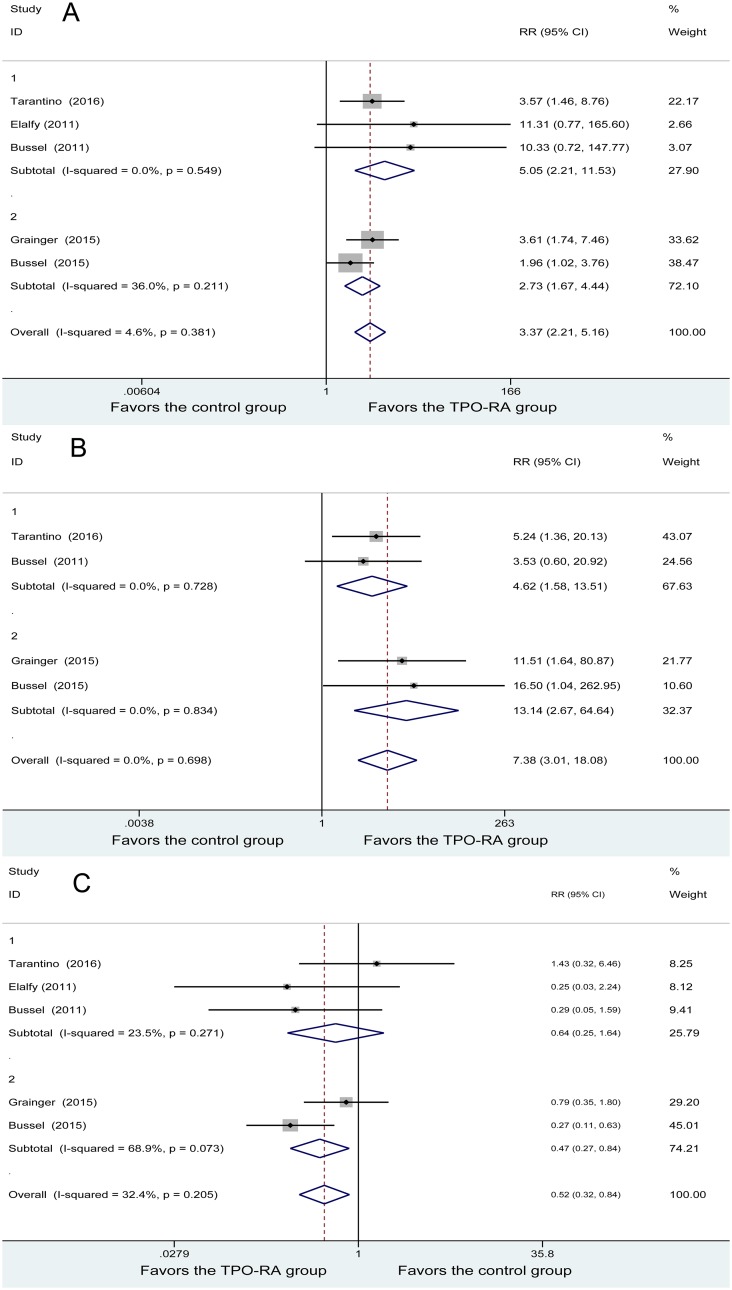
Forest plot of primary outcomes (**A**) overall platelet response. (**B**) Durable response. (**C**) Forest plot of rescue medication. RR: relative risk, CI: confidence interval, 1: romiplostim, and 2: eltrombopag.

An overall platelet response (OR) was conducted in five studies. No heterogeneity was found among the studies (*P* = 0.381, I^2^ = 4.6%). The OR of 179 patients in the TPO-RA group and 82 patients in the control group was analyzed using a fixed-effects model. The result showed a difference in OR between the two groups (RR = 3. 37, 95% CI (2.21, 5.16), *P* = 0). Subgroup meta-analysis based on TPO-RA regimens demonstrated that both romiplostim and eltrombopag were associated with higher rates of OR (RR = 5.05, 95% CI (2.21, 11.53); RR = 2.73, 95% CI (1.67, 4.44), respectively). In short, the TPO-RA group had a higher rate of OR (Figure 2A).

Durable response (DR) was conducted in four studies. The analysis of DR showed no heterogeneity among the studies (*P* = 0.698, I^2^ = 0%). DRs of 167 patients in the TPO-RA group and 76 patients in the control group were analyzed using a fixed-effects model, and a significant difference was found between the two methods (RR = 7.38, 95% CI (3.01, 18.08), *P* = 0). Subgroup meta-analysis based on TPO-RA regimens demonstrated that both romiplostim and eltrombopag were associated with higher rates of DR (RR = 4.62, 95% CI (1.58, 13.51); RR = 13.14, 95% CI (2.67, 64.64), respectively). In brief, the TPO-RA group had a higher rate of DR (Figure 2B).

Rescue medication was conducted in four studies. The analysis of rescue medication showed no heterogeneity among the studies (*P* = 0.205, I^2^ = 32.4%). Rescue medication of 179 patients in the TPO-RA group and 82 patients in the control group were analyzed using a fixed-effects model, and a significant difference was found between the 2 methods (RR = 0.52, 95% CI (0.32, 0.84), *P* = 0.008). Subgroup meta-analysis based on TPO-RA regimens demonstrated that eltrombopag was associated with lower rates of rescue medication needed (RR =0.64, 95% CI (0.25, 1.64), *P* = 0.010), while no great difference was found between romiplostim and the control group (RR = 0.47, 95% CI (0.27, 0.84), *P* = 0.356). In brief, the TPO-RA group had a lower rate of rescue medication needed (Figure 2C).

### Bleeding events

**Figure 3:**
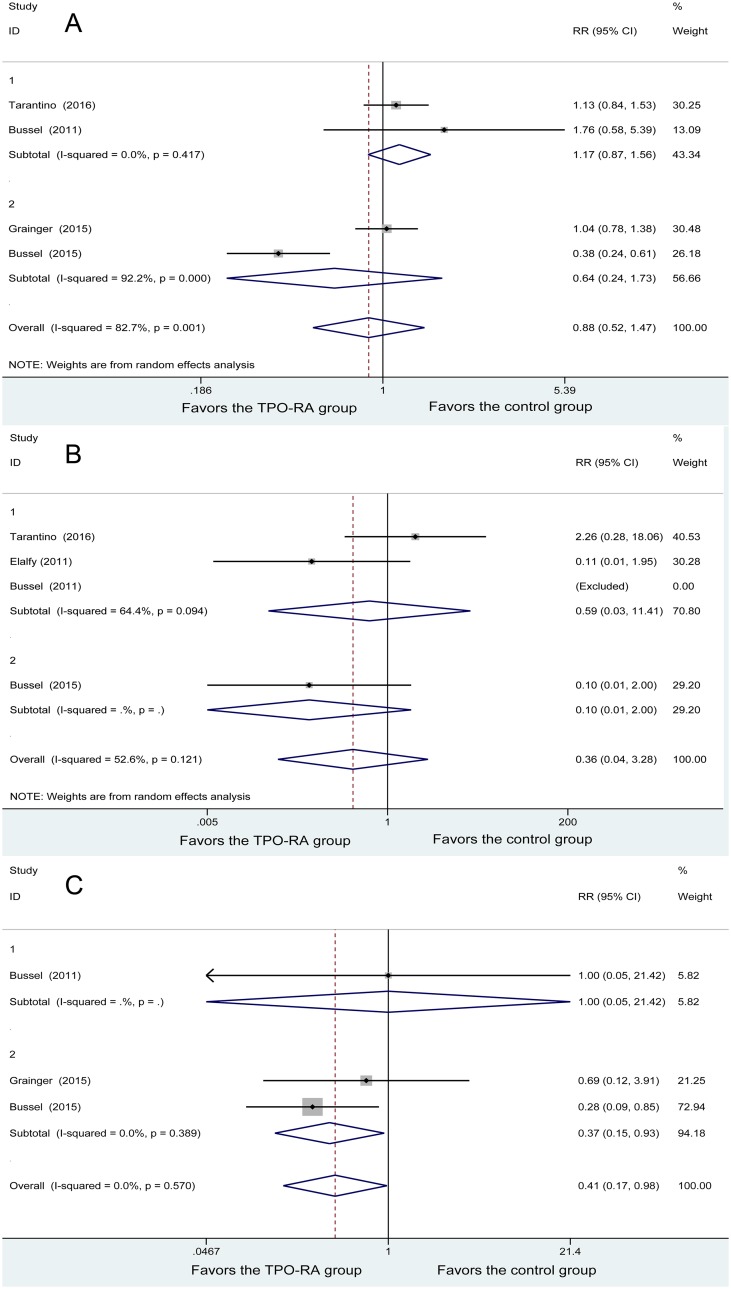
Forest plot of bleeding events (**A**) Any bleeding events. (**B**) Severe bleeding events. (**C**) Clinically significant bleeding events.

Four studies included any bleeding events, and heterogeneity was found among the studies (*P* = 0.001, I^2^ *=* 82.7%). The 167 patients in the TPO-RA group and 75 patients in the control group were analyzed using a random-effects model. No significant difference was found between the 2 groups (RR = 0.88, 95% CI (0.52, 1.47), *P* = 0.620). Subgroup meta-analysis based on TPO-RA regimens demonstrated that both romiplostim and eltrombopag did not differ with the control group in any bleeding events (RR = 1.17, 95% CI (0.87, 1.56); RR = 0.64, 95% CI (0.24, 1.73), respectively) (Figure 3A).

Four studies demonstrated severe bleeding events, with heterogeneity being found among the studies (*P* = 0.121, I^2^ = 52.6%). Using the random-effects model, 116 patients in the TPO-RA group and 52 patients in the control group were analyzed, and no significant difference was found in the severe bleeding events (RR = 0.36, 95% CI (0.04, 3.28), *P* = 0.366). Subgroup meta-analysis based on TPO-RA regimens demonstrated that both romiplostim and eltrombopag had no significant difference with the control group in severe bleeding events (RR = 0.59, 95% CI (0.03, 11.41); RR = 0.10, 95% CI (0.01, 2.00), respectively) (Figure 3B).

Three studies demonstrated clinically significant bleeding events, with no heterogeneity being found among the studies (*P* = 0.570, I^2^ = 0%). Using the fixed-effects model, 125 patients in the TPO-RA group and 56 patients in the control group were analyzed, and a significant difference was found between the two methods (RR = 0.41, 95% CI (0.17, 0.98), *P* = 0.008). Subgroup meta-analysis based on TPO-RA regimens demonstrated that eltrombopag was associated with lower rates of clinically significant bleeding events (RR = 0.37, 95% CI (0.15, 0.93), *P* = 0.035), while no great difference was found between romiplostim and the control group (RR = 1.00, 95% CI (0.05, 21.42), *P* = 1). In brief, the TPO-RA group had a lower rate of clinically significant bleeding events (Figure 3C).

### Second outcomes

**Figure 4:**
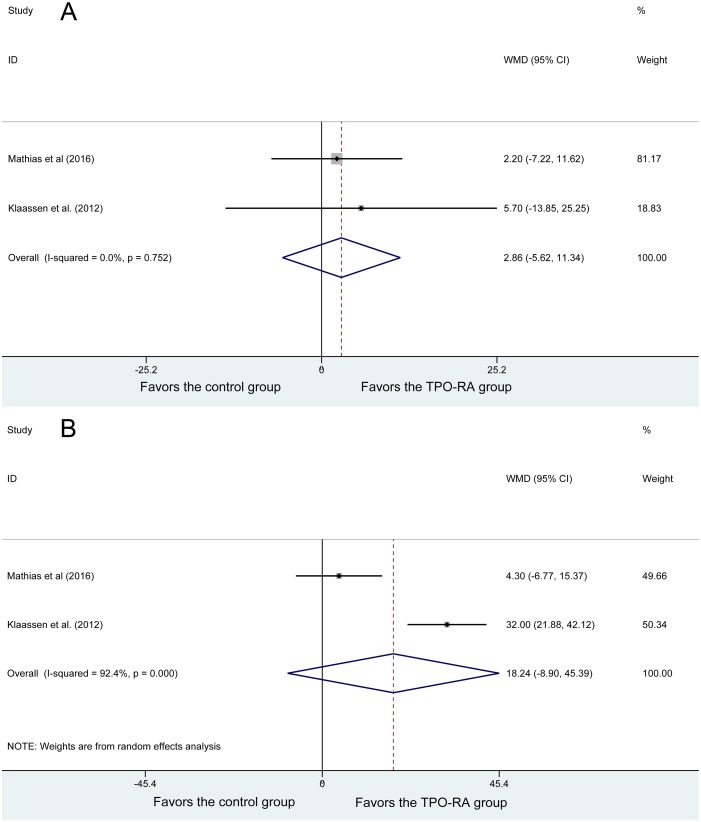
Forest plot of second outcomes (**A**) Health-related quality of life. (**B**) Parental burden.

Two studies reported HRQoL. No heterogeneity was found among the studies (*P* = 0.752, I^2^ = 0%). Using the fixed-effects model, 59 patients in the TPO-RA group and 25 patients in the control group were analyzed, and no significant difference in the HRQoL; WMD = 2.86, 95% CI (−5.62, 11.34), *P* = 0.509 (Figure 4A).

Two studies reported parental burden, and heterogeneity was found among the studies (*P* = 0, I^2^ = 92.4%). Using the random-effects model, 59 patients in the TPO-RA group and 25 patients in the control group were analyzed, and no significant difference was found in parental burden (WMD = 18.24, 95% CI (−8.9, 45.39), P = 0.188) (Figure 4B).

### Safety profiles

**Figure 5:**
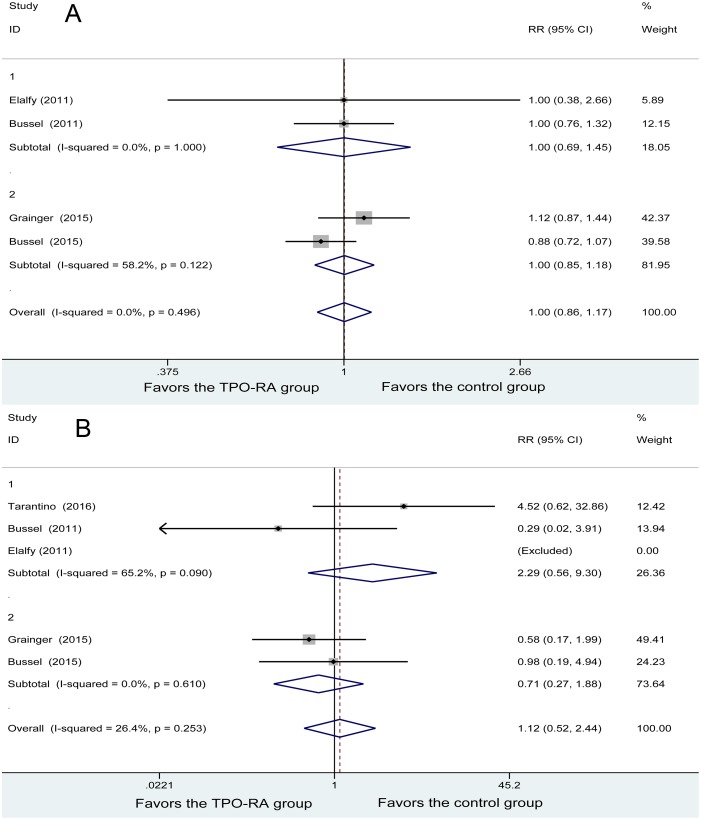
Forest plot of safety profiles (**A**) Any adverse events. (**B**) Severe adverse events.

Four studies reported any adverse events. No heterogeneity was found among the studies (*P* = 0.496, I^2^ = 0%). Using the fixed-effects model in the analysis, with 137 patients in the TPO-RA and 62 patients in the control group, the results showed no great difference in any adverse events between the two groups (RR = 1.00, 95% CI (0.86, 1.17), *P* = 0.974). Subgroup meta-analysis based on TPO-RA regimens demonstrated that both romiplostim and eltrombopag had no significant difference with the control group in any adverse events (RR = 1.00, 95% CI (0.69, 1.45); RR = 1.00, 95% CI (0.85, 1.18), respectively) (Figure 5A).

Five studies reported severe adverse events. No heterogeneity was found among the studies (*P* = 0.253, I^2^ = 26.4%). Using the fixed-effects model, with 179 patients in the TPO-RA group and 81 patients in the control group, the results showed no great difference in severe adverse events between the 2 groups (RR = 1.12, 95% CI (0.52, 2.44), *P* = 0.974). Subgroup meta-analysis based on TPO-RA regimens demonstrated that both romiplostim and eltrombopag had no significant difference with the control group in any adverse events (RR = 2.29, 95% CI (0.56, 9.30); RR = 0.71, 95% CI (0.27, 1.88), respectively) (Figure 5B).

### Publication bias

**Figure 6:**
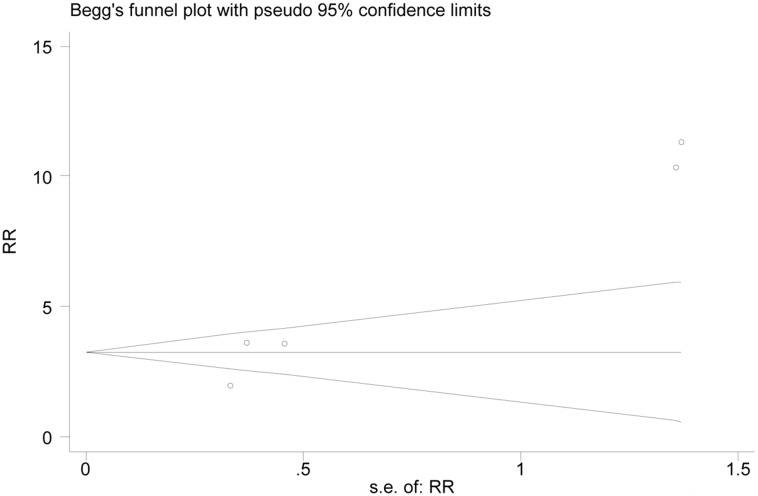
Funnel plot of publication bias for overall platelet response

For OR, which was used as an indicator in most studies as an example, Begg's test was used to assess publication bias. A lack of bias was found among the included studies (Begg's test, *P* = 0.086, Figure 6). At the same time, according to Egger's test, there was a bias among the included studies (Egger's test, *P* = 0.013).

## DISCUSSION

Our recent meta-analysis had several advantages over the previous studies conducted by Wang [6] and Elgebaly [35]. First, more RCTs about pediatric ITP were included in our study. Although there were thirteen RCTs in Wang's study [6] and six RCTs in Elgebaly's [35], only five RCTs in Wang's study [6] and two RCTs in Elgebaly's study [35] were about pediatric ITP. However, we had seven relevant RCTs in this meta-analysis. Second, our study included more outcome parameters, such as clinically significant bleeding events, HRQoL, and parental burden. Third, in our study, subgroup analysis was conducted based on different TPO-RA regimens (eltrombopag or romiplostim) in pediatric ITP patients. In Wang's [6] study, subgroup analysis was conducted between eltrombopag and romiplostim in all ITP patients or between adults and children in the TPO-RA group. Heterogeneity still existed between romiplostim and eltrombopag in pediatric ITP patients. In our opinion, the results of our study are more objective, comprehensive, and characteristic in pediatric ITP patients.

Our meta-analysis provides evidence that TPO-RAs are effective agents in the treatment of childhood chronic ITP. TPO-RAs were found to significantly improve OR and DR, decrease the number of patients requiring rescue treatment, and reduce the incidence of clinically significant bleeding events, although no statistically significant difference was found in the risk of any bleeding events and severe bleeding events. HRQoL and parental burden were newly included outcome parameters but showed no significant difference between the TPO-RA group and control group. Meanwhile, TPO-RAs appeared to be safe in children with chronic ITP. Incidences of any adverse effects and severe adverse effects in the TPO-RA groups were similar to those in the control groups.

In our study, both OR and DR were significantly improved by TPO-RAs in the treatment of chronic ITP in children. Subgroup analysis suggested that both eltrombopag and romiplostim significantly improved the OR and DR. This result was in accordance with studies [6, 13, 35] on the treatment of chronic ITP in adults. TPO-RAs are drugs identical to endogenous TPOs, which can bind and activate the TPO-receptor to mimic TPO activity [5, 11]. Activating platelet production via the TPO pathway, TPO-RAs often provide rapid and durable platelet responses [5, 13, 29–31]. Furthermore, the rate of rescue medication needed decreased in the TPO-RA group. Subgroup analysis suggested that great differences were found between eltrombopag and the control group, but no significant difference was found between romiplostim and the control group. Two included studies [7, 18] found that eltrombopag could significantly reduce incidences of rescue therapy. However, Tarantino's study [4] showed that the use of rescue medication did not differ between the romiplostim and control group. This result contrasts with those of romiplostim studies [1, 34], in which incidences of rescue therapy are significantly low in romiplostim. Eltrombopag appears to be a better TPO-RA in the treatment of chronic ITP in children in terms of the rate of rescue medication needed.

Bleeding can be used as a weekly assessment, according to Common Terminology Criteria for Adverse Events (CTCAE) version 3.0 [36]. In theory, TPO-RAs can reduce the incidence of bleeding by promoting platelet production. In our study, the TPO-RA group had a low incidence of clinically significant bleeding events but comparable results in any bleeding events and severe bleeding events. As a composite bleeding episode endpoint, clinically significant bleeding (CTCAE grade ≥ 2) was a meaningful outcome parameter for efficacy [4]. Subgroup analysis found that eltrombopag could significantly reduce the incidence of clinically significant bleeding, but romiplostim was not different from the control group. The rate of clinically significant bleeding events was significantly low in the two included studies [7, 18], while no difference was found in Bussel's study [1]. Pediatric ITP patients present with a range of bleeding symptoms, from minimal to life-threatening ones. In our opinion, some minimal symptoms may be difficult to diagnosis, and the result of any bleeding events maybe not objective. As for severe bleeding events, 3 included studies [1, 7, 34] showed a low incidence of severe bleeding events, whereas Tarantino's study [4] showed no difference between the 2 groups. Eltrombopag appears to be a better TPO-RA in the treatment of chronic ITP in children, according to the incidence of clinically significant bleeding.

Because of the fear and disturbance associated with bleeding and restriction of daily life, pediatric ITP is a potentially bleeding disease that may also affect a child's HRQoL and parental burden [5]. In our study, although no great difference was found between the two groups in the HRQoL, studies [8, 12] suggested that treatment with romiplostim might be associated with improved HRQoL in children with chronic ITP. Furthermore, the increased frequency of hospital visits may have bothered some children and resulted in low child self-report scores. As for parental burden, significant differences could be found between romiplostim and the control group in both studies. Since there was more homogeneity, the random-effects model was used to analyze the data, which may influence the result. HRQoL and parental burden were newly included outcome parameters, which were used in only two RCTs [8, 12]. Although no difference was found between the TPO-RA group and the control group for the 2 outcome parameters, it might be more meaningful to be included in the future RCTs in the treatment of pediatric ITP.

For the safe profile, the TPO-RA group was not different with the control group in terms of any adverse effects and serious adverse effects. In our study, headache, epistaxis, upper respiratory tract infection and nasopharyngitis are common adverse effects for children [1, 4, 7, 18, 34], while thromboembolic events and amino transferase abnormalities were less, which were relatively common in adult patients [13]. Several open-label extension studies [37–39] found that long-term treatment with romiplostim maintained platelet counts with a safety profile in children with chronic ITP.

Several studies [7, 18, 34] have reported encouraging result for both TPO-RAs; however, no study directly compares the efficacy and safety of the 2 drugs. In our study, eltrombopag appears to be a better TPO-RA in the treatment of chronic ITP in children in terms of the rate of rescue medication needed and the incidence of clinically significant bleeding.

No publication bias was found in the Begg's test. However, there was a bias according to Egger's test. The reason for this was probably that 3 RCTs [40–42] in the form of conference abstracts were excluded because the data were the same as those in the included articles.

The limitations of this study were as follow. First, the sample size was not large and the outcome indicator was not unified, which may have influenced the outcome. Second, the follow-up durations in the studies varied; they may not have been sufficiently homogeneous to evaluate the differences between the two groups. Third, the safety profiles in the included studies could only be evaluated during a short-term follow-up where some chronic complications might have been ignored.

In conclusion, this meta-analysis demonstrated that TPO-RAs are effective and safe agents for the treatment of chronic ITP in pediatric patients. Eltrombopag appears to be better than romiplostim in terms of the rate of rescue medication needed and clinically significant bleeding.

## MATERIALS AND METHODS

### Identification of eligible studies

PubMed, Cochrane Library, and Embase databases were searched from their earliest entries up to June 2017. A manual search of all reference lists contained in the literature was also performed. The search strategy was (((((“romiplostim” [Supplementary Concept]) OR “eltrombopag” [Supplementary Concept]) OR ((((thrombopoietin receptor agonists[Title/Abstract]) OR TPO-Ras[Title/Abstract]) OR Romiplostim[Title/Abstract]) OR Eltrombopag[Title/Abstract]))) AND ((((((immune thrombocytopenia[Title/Abstract]) OR idiopathic thrombocytopenia purpura[Title/Abstract]) OR ITP[Title/Abstract]) OR Autoimmune thrombocytopenia[Title/Abstract])) OR “Purpura, Thrombocytopenic, Idiopathic”[Mesh])) AND ((((Randomized controlled trial[Publication Type]) OR Random*[Title/Abstract]) OR “Randomized Controlled Trials as Topic”[Mesh])).

### Inclusion and exclusion criteria

The inclusion criteria were as follows: (I) subject—all pediatric patients with a confirmed diagnosis of chronic ITP, with no limitation regarding sex or race; (II) intervention method—comparison of clinical outcome between the TPO-RA group and the control group in the therapy; (III) outcome parameters—OR; DR; rescue medication; any bleeding events; severe bleeding events; clinically significant bleeding events; all adverse effects; severe adverse effects; (IV) study type—prospective randomized controlled trial (RCT).

The exclusion criteria were as follows: (I) nonprospective trials (e.g., retrospective studies, observational studies, case series, and reviews); (II) studies about adult patients; (III) comparisons that were not about the TPO-RA group and the control group in the therapy; (IV) studies with low levels of evidence; and (V) laboratory studies.

### Literature selection

All potential studies were imported into Endnote X7, and duplicates were excluded. Then, two researchers independently excluded studies based on titles and abstracts. Last, by reading the full text carefully, the two researchers eliminated the studies that did not satisfy the selection criteria. Disagreements were resolved by discussion with the corresponding researcher.

### Quality assessment

Two researchers selected articles independently according to the criteria above and assessed the quality of each. All disagreements were resolved by the corresponding researcher. The Physiotherapy Evidence Database (PEDro) scale [33], which consists of 11 items based on the Delphi list, was used to assess the methodological quality of each article. Each item was scored yes or no, with a maximum score of 10 because the first criterion was not scored. A trial with a score of ≥ 6 was considered to be of high quality.

### Data extraction

Two researchers independently extracted the data from the articles using the same format, after which the data were compared and disagreements were resolved by extracting and reviewing the data again, including information about the study, such as article information (author and publication date), participant demographics, follow-up period, sample size, baseline platelet count, duration of ITP, splenectomy, and outcome parameters.

### Statistical methods

The meta-analysis was conducted using Stata/SE version 12.0. All extracted data were checked and inputted by reviewers. When the outcome indicator was dichotomous outcomes, relative risk (RR) was calculated for effect size. For continuous outcomes, weighted mean difference (WMD) was calculated when the same measurement criterion was used; otherwise, standardized mean difference (SMD) was calculated; both used 95% confidence intervals (CI). The intervening effect of an indicator was considered a zero difference if 95% CI for WMD or SMD contained 0 and 95% CI for RR contained 1. The statistical heterogeneity was tested with the chi-square test and I^2^. If heterogeneity was low (*P* > 0.1 or I^2^ ≤ 50%), a fixed-effects model was used. If heterogeneity was significant (*P* < 0.1, I^2^ > 50%), sensitivity analysis, subgroup analyses, and meta-regression were conducted to find the source of the heterogeneity. If the heterogeneity could not be eliminated, a random-effects model would be used when the meta-analysis results had clinical homogeneity. Otherwise, descriptive analysis would be used. Begg's test was used to check the publication bias of the involved articles.
